# Oxidation of Long-Chain α-Olefins Using Environmentally-Friendly Oxidants

**DOI:** 10.3390/ma13204545

**Published:** 2020-10-13

**Authors:** Kamil Peckh, Dawid Lisicki, Gabriela Talik, Beata Orlińska

**Affiliations:** Department of Organic Chemical Technology and Petrochemistry, Silesian University of Technology, B. Krzywoustego 4, 44-100 Gliwice, Poland; kamil.peckh@polsl.pl (K.P.); dawid.lisicki@polsl.pl (D.L.); gabriela.talik@polsl.pl (G.T.)

**Keywords:** α-olefins, oxidation, oxidative cleavage, *N*-hydroxyphthalimide

## Abstract

Studies on the oxidation of α-olefins via the two-stage method are presented. The new method consisted of oxidizing C30+ α-olefins with hydrogen peroxide (2 equiv.) and subsequent oxidation with oxygen. Products with high acid numbers (29–82 mgKOH/g) and saponification numbers (64–140 mgKOH/g) were obtained and compared with products obtained using only hydrogen peroxide or oxygen. It was demonstrated that H_2_O_2_ can be partially replaced by oxygen in the oxidative cleavage reaction of α-olefins. *N*-hydroxyphthalimide in combination with Co(acac)_2_ demonstrated high activity in the oxidation stage using oxygen.

## 1. Introduction

**Scheme 1:**
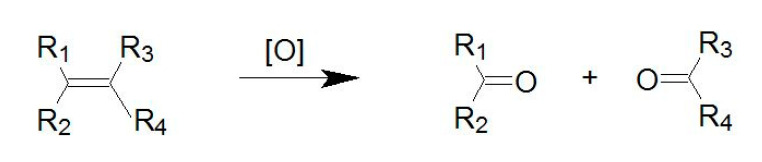
Oxidative cleavage of olefins.

The oxidative cleavage of alkenes is an important type of reaction in the chemical industry. In such reactions, an oxidizing agent cleaves C=C double bonds (Scheme 1) to produce two carbonyl-containing compounds (aldehydes and/or ketones). Aldehydes can be further oxidized to corresponding carboxylic acids. These are well-studied reactions, and numerous data has been produced on the use of various oxidants, catalysts, and raw materials. In addition to alkenes, terpenes, oils, fats, and fatty acid derivatives containing double bonds have been used as raw materials [1].

Various oxidizing agents, including KMnO_4_ [2,3] and chromium (e.g., CrO_2_Cl_2_ [4]), ruthenium (e.g., RuO_4_ [5]), and osmium (e.g., OsO_4_ [6]) oxides have been studied. These oxidants are often used in stoichiometric amounts and combined with other oxidants [1], e.g., OsO_4_ was used in a mixture with NaIO_4_ (the so-called Lemieux-Johnson protocol). Unfortunately, ruthenium and osmium are expensive metals, and osmium and chromium are also very toxic, which limits the industrial application of the above-mentioned systems [1]. Oxone [7,8], PhI(OAc)_2_ [9], *m*-chloroperoxybenzoic acid [10], and *tert*-butyl hydroperoxide [11,12,13,14,15] have also been used as oxidants. Industrial oxidative cleavage reaction of oleic acid to pelargonic and azelaic acids [1] is carried out using ozone as an oxidizing agent without the addition of a metallic catalyst [16]; however, due to its instability, ozone is hazardous and requires *in situ* formation, and it also requires specialized equipment, which increases production costs [17].

For several years, much attention has been devoted to the use of ecologically-friendly oxidizing agents such as hydrogen peroxide and oxygen in oxidative cleavage reactions [18]. Oxygen is used because of its lower cost, which is particularly important for large-scale processes. Unfortunately, oxygen works well only in case of olefins containing a phenyl ring at the vinyl position, and the use of oxygen often requires specialized or expensive catalytic systems [19,20]. 

**Scheme 2:**
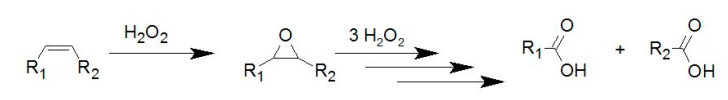
Tungsten-catalyzed oxidative cleavage reaction of aliphatic olefins using hydrogen peroxide.

The use of H_2_O_2_ in the oxidative cleavage of aliphatic olefins [21,22,23,24,25,26,27,28] has been widely described. H_2_O_2_ is most often used in combination with metallic catalysts based on selenium [29], palladium [30], rhenium [21], ruthenium [31], iron [22], and especially tungsten [23,24,25,26,27,32,33,34,35,36]. Tungsten-catalyzed reactions proceed through a mechanism that involves epoxidation, epoxide hydrolysis to diol, and subsequent oxidation of the diol to the corresponding aldehyde, which may be subsequently oxidized to a carboxylic acid (Scheme 2). Tungsten was introduced into the system in the form of H_3_PW_12_O_40_ [25,36], Na_2_WO_4_ [24,27,33], and most often as H_2_WO_4_ [28,34,35]. 

When hydrogen peroxide is used, a two-phase system is formed, making it necessary to use a phase-transfer catalyst, which is most often a tetraalkylammonium salt [23,24,25,26,27,28,32,33,36]. As a result of the interactions between hydrogen peroxide, tungsten compounds, and phase transfer agents, new, more active catalysts are often formed. For example, a catalyst [C_5_H_5_N(CH_2_)_15_Me]_3_{PO_4_[WO(O_2_)_2_]_4_} was obtained using H_2_O_2_, H_3_PW_12_O_40_·15.4·H_2_O, and [C_5_H_5_N(*n*-C_16_H_33_)][Cl] to oxidatively cleave oleic acid [32]. After 1 h of reaction at 80 °C, azelaic and pelargonic acids were obtained in yields of 86% and 82%, respectively. 

Undoubtedly, the advantages of using H_2_O_2_ as an oxidant are the formation of water as a by-product and the high content of active oxygen (47 wt.%) [37]. Disadvantages include the introduction of large amounts of water into the system due to its concentration—in the range of 30–60%. In addition, according to the reaction stoichiometry, it is necessary to use 4 equiv. of H_2_O_2_, and in the processes described in the literature, H_2_O_2_ is often used in stoichiometric excess. For example, as much as 325 mmol of H_2_O_2_ per 65 mmol of raw material was used in the oxidation of oleic acid, which is 5 equiv. of this oxidant [36]. 

**Scheme 3:**
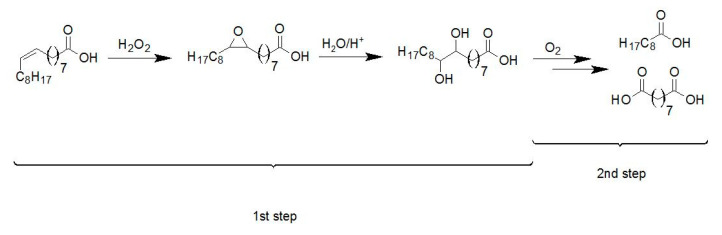
Two-step oxidative cleavage of oleic acid.

According to the limited literature data, hydrogen peroxide can be partially replaced in this process with cheaper and abundant oxygen. Hydrogen peroxide is necessary for the epoxidation of carbon-carbon double bonds, while oxygen can be used in subsequent oxidation stages of formed diol by epoxide hydrolysis. So far, such a solution has been reported for the oxidation of oleic acid to azelaic and pelargonic acids (Scheme 3) [38,39,40].

The reported processes involve epoxidation catalyzed by H_2_WO_4_ followed by hydrolysis to a diol [38,39,40]. In the second stage, in situ formed catalysts composed of H_2_WO_4_ and Co(OAc)_2_ or polyoxometalates such as H_6_CoW_12_O_40_ and (NH_4_)_8_[Co_2_W_12_O_42_] [38,39] as well as Co(acac)_3_ in combination with *N*-hydroxyphthalimide (NHPI) have used [40]. Oakley [40] introduced the catalysts NHPI/Co(III) directly into the product of the first stage obtained by oxidation of oleic acid by H2O2 (only 1.2 mmol H2O2/mol C=C) in the presence of H2WO4 in *tert*-butanol at reflux (2 h). Aerobic oxidation was carried out for 3 h at 70–75 °C, which produced azelaic and pelargonic acids in 15% yield. Higher yields were obtained when methyl erucate was used as the raw material, and brassylic and pelargonic acids were obtained in 41% and 54% yields, respectively.

The high catalytic activity of NHPI has been demonstrated in many free-radical oxidation reactions using oxygen, including the aerobic cleavage of alkenes. α-Methylstyrene and its derivatives were oxidized to acetophenones by oxygen in the presence of NHPI combined with cobalt(II) compounds, azo compounds, or alkylammonium salts [41]. Unfortunately, the cleavage of double bonds of non-aromatic alkenes, such as 1-cyclohexene or 1-dodecene, did not occur. 

Herein, we describe the oxidation of long-chain α-olefins (C30+), consisting of subsequent oxidation by hydrogen peroxide and oxygen. We have recently described the oxidative cleavage of C30+ alkenes using 5 equiv. of H_2_O_2_ and catalyst composed of H_2_WO_4_, Luviquat as PTC and H_2_SO_4_ at 90 °C [28]. This paper investigates the possibility of partially replacing H_2_O_2_ with cheaper oxygen in this process. To the best of our knowledge, such a two-stage oxidation process for long-chain, linear α-olefins has not yet been described. Long-chain olefins were obtained as a product of ethylene oligomerization, thermal cracking of paraffins, or polyethylenes. The significance of the latter method may be higher due to the possibility of using post-consumer polyethylene as a raw material. Polar products obtained by the oxidation of long-chain olefins containing carboxyl and ester groups can be used to produce surfactants, plasticizers, waxes, lubricants, greases, and adhesives. 

## 2. Materials and Methods 

### 2.1. Materials

Alpha Plus C30+ olefins (Chevron-Phillips, Diegem, Belgium), hydrogen peroxide (30 or 50 wt.% in water, Acros Organics), tungstic acid (Fluka), cetyldimethyl(2-hydroxyethyl)ammonium dihydrogen phosphate (Luviquat, 30% in water, Aldrich, Steinheim, Germany), *N*-hydroxyphthalimide (NHPI, Sigma-Aldrich, Steinheim, Germany), Co(acac)_2_ (Sigma-Aldrich), and methylcyclohexane (MCH, Alfa Aesar, Karlsruhe, Germany) were commercially available and used without purification.

### 2.2. C30+ Olefins Oxidation Using H_2_O_2_

C30+ α-olefin (40 or 200 g), H_2_WO_4_ (0.75–3.0 wt.%), Luviquat (0.75–5.0 wt.%), 0.5 M H_3_PO_4_ or 0.5 M H_2_SO_4_ (0–0.1 cm^3^), and (optional) methylcyclohexane as diluent were placed in a round bottom flask equipped with a mechanical stirrer (300–400 rpm) and heated to 70 °C. Next, hydrogen peroxide (30 or 50 wt.%) was dropped (2 g/min), and the mixture was heated to 85 °C. The reaction was conducted at 85 °C for 2–10 h. Products were purified by washing once at 80–90 °C with distilled water (100 or 500 mL, resp.) and dried at 80 °C. Next, acid numbers (AN) and saponification numbers (SN) of products were determined.

### 2.3. C30+ Olefins Oxidation Using H_2_O_2_ and O_2_

C30+ α-olefin (40 or 200 g) were oxidized using hydrogen peroxide, purified by washing with water and dried according to the described above method. The obtained product (15 g), Co(acac)_2_ (0.1–1 wt.%), and/or NHPI (1 wt.%) were introduced into a 100 mL reactor (Autoclave Engineers Inc., Clevelend, OH, USA) made of Hastelloy C-276 steel and equipped with a mechanical stirrer, heating jacket, temperature and pressure sensors, and reflux condenser. The mixture was heated to 90 °C to melt the wax, then the reactor was sealed and filled with oxygen up to a pressure of 0.5 MPa and then heated up to reaction temperature. The reaction was carried out at 90–110 °C for 2.5–7.5 h at 1000 rpm. The AN and SN of the obtained product were determined.

The reaction at atmospheric pressure using an oxygen flow rate of 3 L/h was also carried out in the above-described autoclave (100 °C, 0.1 MPa, 5 h, 1000 rpm).

### 2.4. C30+ Olefins Oxidation Using O_2_

C30+ α-olefin (15 g), Co(acac)_2_ (0.1 wt.%) and/or NHPI (1 wt.%) were introduced into a 100 mL above-described reactor (Autoclave Engineers Inc., USA) and oxidized under an oxygen pressure of 0.5 MPa at 100 °C for 5 h at 1000 rpm, as described above. The AN and SN of the obtained product were determined.

### 2.5. Double-Bond Content Calculation

The double-bond content in the starting material and obtained products was calculated based on ^1^H NMR spectroscopy (Agilent, Palo Alto, CA, USA). Sample (0.01–0.02 g) and naphthalene (0.001–0.002 g) as an internal standard were dissolved in CDCl_3_ and introduced into NMR tube. The ratio of the number of double-bond to the number of naphthalene molecules was calculated by integration of the peak areas corresponding to the respective functional groups. 

### 2.6. Acid Number

A sample (ca. 0.3 g) of the products and mixture of 25 mL of xylene with 2-methyl-2,4-pentanediol (2:1 *v/v*) were placed in a conical flask equipped with a condenser. The mixture was heated to obtain a clear solution. Next, the hot mixture was titrated with 0.05 M KOH in ethanol using phenolphthalein as an indicator. The acid number (AN) is the mass of KOH (in mg) required to neutralize the carboxylic acid groups in 1 g of sample.

### 2.7. Saponification Number

A sample (ca. 1 g) of the product, a mixture of 25 mL of xylene with 1-propanol (4:1 v/v) and 25 mL of 0.1 M KOH in ethanol were placed in a conical flask. The mixture was heated under reflux for 30 min. Next, the hot mixture was titrated with 0.1 M HCl using phenolphthalein as an indicator. In a blank test, the same mixture but without the sample was used. The saponification number (SN) is the mass of KOH (in mg) required to neutralize the free carboxylic groups and esters in 1 g of sample.

### 2.8. Viscosity Determination

Dynamic viscosity was determined using Brookfield Rheometer (Brookfield Inc., Toronto, ON, Canada). A sample (ca. 2.5 g) of the product was put on a measuring plate heated with circulating oil at a set temperature of 120 °C. Then, the measurements were performed using five preset shear rates (200–4000 s^−1^). The dynamic viscosity of products and raw material was constant in the given range of shear rates, showing that they behaved as Newtonian fluids.

### 2.9. Apparatus

The following apparatus were used in this study: a gas chromatograph (GC, Agilent Technologies 7890C, (Agilent, Palo Alto, CA, USA) equipped with a ZB 5HT column (30 m × 0.25 mm × 0.25 μm) connected to a mass spectrometer (MS, Agilent Technologies 5975C; EI 70 eV); an EZ-Melt MPA120 automated melting point apparatus with digital image processing software (Stanford Research Systems, Sunnyvale, CA, USA); Brookfield RST CPS Rheometer (Brookfield Inc., Toronto, ON, Canada) with RCT-75-1 and RCT-25-2 and Rheo3000 software; an FT-IR Mettler-Toledo iC10 spectrometer (Mettler-Toledo, Columbus, OH, USA) equipped with an ATR probe; an Agilent 400 NMR spectrometer operating at 400 MHz.

## 3. Results and Discussion

**Figure 1:**
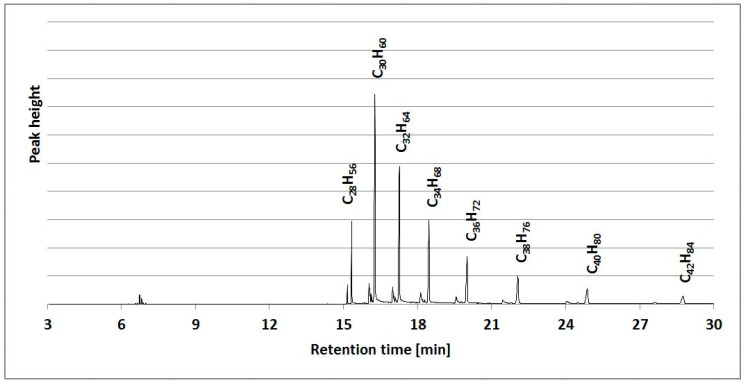
GC-MS chromatogram of starting material C30+ olefins (mass spectra Figures S1–S8).

**Figure 2:**
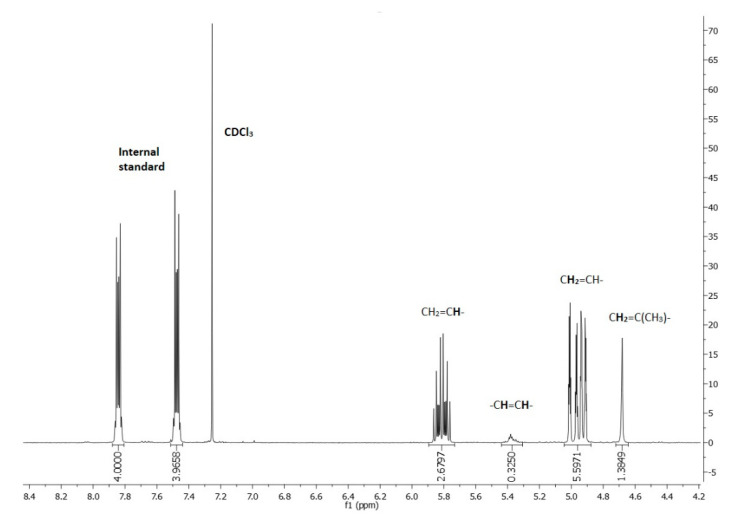
^1^H NMR spectrum of starting material C30+ olefins.

Studies on the oxidation of α-olefins via a two-stage method were carried out. The method consisted of oxidizing the raw material with hydrogen peroxide (first stage) and subsequent oxidation of the product with oxygen (second stage). For comparison, olefins were also oxidized using only H_2_O_2_ or oxygen. Commercially-available C30+ olefins were used as the starting material (C30+). According to the producer (Chevron-Phillips), they contain 71.4 wt.% of linear and 24.7 wt.% of branched α-olefins. GC-MS analysis showed that the C30+ olefins is a mixture of olefins containing an even number of carbon atoms (mostly C28 to C42) (Figure 1) [28]. Based on the surface areas, the amount of hydrocarbons in the raw material increased in the following order: C30 > C32 > C28 > C34 > C36 > C38 > C40 > C42. The double bond content in C30+ olefins determined based on ^1^H NMR spectroscopy was 2.030 mmol C=C/g, corresponding to the theoretical bromine number of 32.44 g Br_2_/100 g) (Figure 2). The viscosity of C30+ was 3.7 mPa∙s (at 120 °C), and the melting point was in the range of 59.6–69.6 °C. The oxidation degree of obtained products was established based on the content of carboxyl and ester groups by determining their acid number (AN) and saponification number (SN). For selected products, their viscosity, double bond content, and composition using GC-MS were also determined.

### 3.1. First Stage: Oxidation of C30+ Olefins Using H_2_O_2_

Based on our previous work [28], the catalyst system composed of H_2_WO_4_, a halogen-free lipophilic PTC, i.e. cetyldimethyl(2-hydroxyethyl)ammonium dihydrogen phosphate (Luviquat), and H_2_SO_4_ or H_3_PO_4_ was chosen for oxidation stage with hydrogen peroxide. The reaction temperature was limited by the thermal stability of hydrogen peroxide and also by the melting point of the raw material and product (85 °C).

The effects of the amount and concentration of hydrogen peroxide, the amount of catalysts, reaction time, and the addition of solvent on the oxidation of C30+ olefins were established in the presented studies.

#### 3.1.1. Effect of the Amount and Concentration of H_2_O_2_

The results of the effect of 30 or 50% H_2_O_2_ on the C30+ olefins oxidation are presented in Table 1. Hydrogen peroxide in amount of 1, 2, 3, and 4 equiv. was used. The reactions were carried out without solvent, as well as in methylcyclohexane, against H_2_WO_4_ (1.5 or 3%), Luviquat (2.5 or 5%), and H_2_SO_4_, at 85 °C for 10 h.

The results showed that increasing the amount of hydrogen peroxide oxidant from 1 to 4 equiv. decreased the number of double bonds in the products. According to the reaction stoichiometry, 1 equiv. of H_2_O_2_ is sufficient for the complete epoxidation of the C=C double bond. However, in our study, using 1 equiv. of H_2_O_2_ resulted in the incomplete conversion of C=C bonds. For example, the conversion was 61% (Table 1, Entry 1) or 55% (Table 1, Entry 13) in a reaction carried out without solvent and in MCH, respectively. This occurred because hydrogen peroxide was consumed in the subsequent oxidation of diols to carboxylic acids, and also because it partially decomposed during the reaction. A two-fold increase in the H_2_O_2_ amount significantly increased the conversion of C=C bonds to 89 and 80%, respectively (Table 1, Entries 2 and 14). The use of H_2_O_2_ at both concentrations, 30 and 50%, provided comparable conversions of C=C bonds. For example, when 2 equiv. of 30 or 50% H_2_O_2_ were used, the conversions reached 84 and 89%, respectively. (Table 1, Entries 10 and 2). The use of 30% H_2_O_2_ is cheaper and safer to use, but it introduces excess water into the system.

It was demonstrated that the addition of MCH only slightly reduced the conversion of C=C bonds (at 2 equiv. H_2_O_2_, conversion decreased from 89 to 80%), while it reduced the reaction mixture viscosity, which facilitated heat removal and thus improved process safety; however, the use of MCH requires additional product purification steps.

In the oxidation of olefins with hydrogen peroxide, an epoxide is first formed, which is then hydrolyzed to the respective diol. In the subsequent reactions, the diol is oxidized to corresponding carboxylic acids, which can form esters with these diols. As expected, the content of carboxylic acids and respective esters in the reaction products increased with the amount of hydrogen peroxide. It was also observed that the product viscosity slightly increased with the oxidation degree.

#### 3.1.2. Effect of Amount of Catalysts

The effect of H_2_WO_4_, Luviquat (Table 2), and H_2_SO_4_ or H_3_PO_4_ (Table 3) on the oxidation of C30+ olefins with hydrogen peroxide was investigated. Reactions were carried out at 85 °C without solvent for 10 h.

The results showed that to obtain a low content of double bonds, it is helpful to use a combination of H_2_WO_4_ and Luviquat (Table 2, Entries 1, 2, 7). Increasing the Luviquat content from 0.75 to 2.5% (Table 2, Entries 5–7) increased the double bond conversion and increased the amount of acids and esters in the products. This, in turn, increased the viscosity of the product. When the amount of H_2_WO_4_ increased from 0.75 to 1.5%, the conversion of C=C bonds also increased, as did the viscosity of the products (Table 2, Entries 7 and 9).

The addition of H_2_SO_4_ or H_3_PO_4_ to the studied system did not affect the double bond conversion, but it increased the content of acids and esters in the products (Table 3). This probably occurred because of their effect on the hydrolysis of the epoxide to a diol, which underwent subsequent oxidation and esterification.

#### 3.1.3. Effect of Time of Reaction

The effect of the reaction time (2–10 h) on the oxidation of C30+ olefins by hydrogen peroxide (2 equiv.) under solvent-free conditions or in MCH as solvent was studied (Table 4).

When the reaction was carried out with 2 equiv. of H_2_O_2_, significant C=C bond conversion was obtained just after 2 h (e.g., against 2.5% Luviquat and 1.5% H_2_WO_4_, it was 91%). Prolonging the reaction time only slightly increased the ester number.

### 3.2. Second Stage: Oxidation Using O_2_

A system composed of NHPI and Co(acac)_2_, known for its high activity for hydrocarbon oxidation with oxygen, was chosen as catalysts for this oxidation step. To determine the effects of catalyst and reaction conditions, we used C30+ olefins pre-oxidized with hydrogen peroxide as the raw material. Its properties are given in Table 5.

#### 3.2.1. Effect of Catalyst

In Table 6, the products obtained by oxidation with oxygen catalyzed by Co(acac)_2_, NHPI, or NHPI/ Co(acac)_2_ system are compared.

It was found that the oxidation reaction did not proceed without the addition of a catalyst. A slight decrease in the AN, SN, and melting point under non-catalytic reaction conditions (Table 6, Entry 2) indicates thermal degradation of the raw material.

As expected, the system consisting of NHPI and Co(II) showed high catalytic activity, and a significant increase in the AN and SN of the product was achieved. At the same time, however, the melting point decreased, which may indicate that products with lower molecular weights were formed by oxygen oxidation. 

Unfortunately, the use of cobalt salts as a catalyst adversely affects the color of the product, which may limit its potential applications; therefore, in terms of product quality regarding the color and metal content, it is more beneficial to use only the organocatalyst NHPI.

#### 3.2.2. Effect of Temperature and Reaction Time

Oxidation reactions using oxygen were carried out at 90–110 °C for 2.5, 5, or 7.5 h in the presence of a catalyst composed of NHPI and Co(II) (Table 7).

It was found that with increasing reaction temperature and time, the obtained products were characterized by increasingly higher AN and SN. At the same time, a lower and broader melting point range was obtained, which might indicate product degradation.

#### 3.2.3. Comparison of C30+ Olefin Oxidation Products Using H_2_O_2_, O_2_, or Both H_2_O_2_ and O_2_

The products obtained by two-step oxidation of C30+ olefins using H_2_O_2_ (1st stage; 2 equiv.) and then O_2_ (2nd stage) as oxidizing agents were compared with products obtained using only H_2_O_2_ (5 equiv.; [28]) or O_2_. Table 8 presents the degree of oxidation of the products based on the AN and SN.

**Figure 3:**
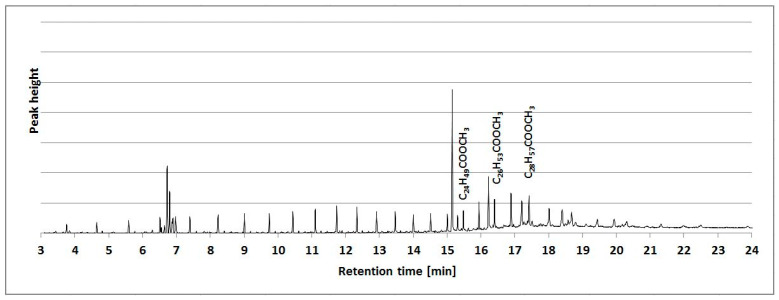
GC-MS chromatogram of product obtained by C30+ olefin oxidation with O_2_ (Table 8, Entry 3; mass spectra Figures S9–S12).

**Figure 4:**
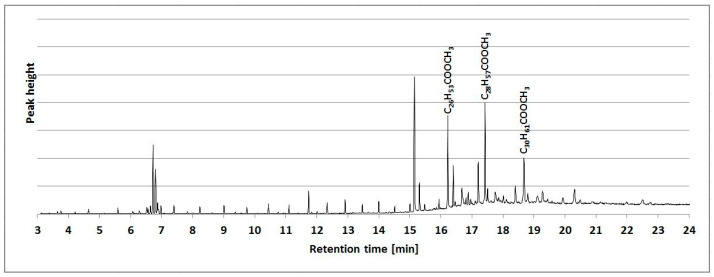
GC-MS chromatogram of product obtained by C30+ olefin oxidation using 2 equiv. of H_2_O_2_ (Table 8, Entry 1a; mass spectra Figures S9–S12).

**Figure 5:**
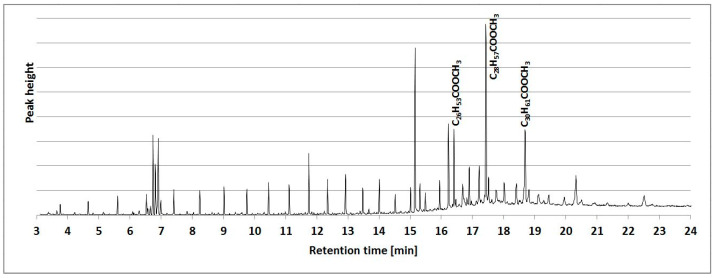
GC-MS chromatogram of product obtained by C30+ olefin oxidation using H_2_O_2_ and O_2_ (Table 8, Entry 1b; mass spectra Figures S9–S12).

**Figure 6:**
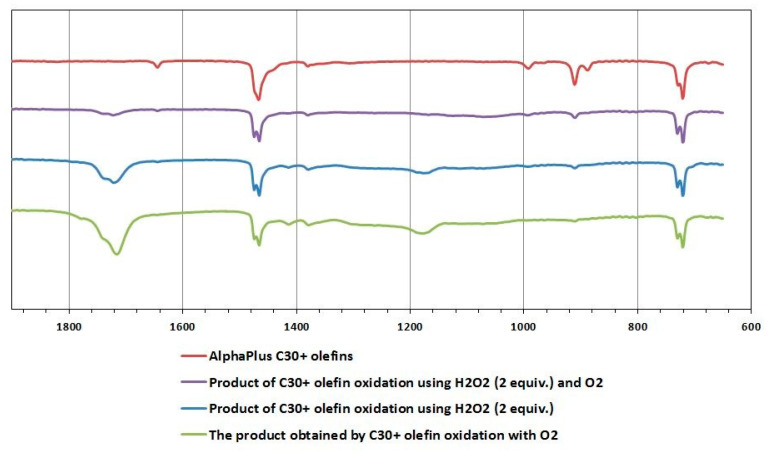
Comparison of FT-IR spectra of oxidation products.

Products with high oxidation degrees were obtained by oxidizing C30+ using only H_2_O_2_ (Table 8, Entry 2) or O_2_ (Table 8, Entry 3), as well as both oxidants (Entry 1). The composition of these products were compared using GC-MS (Figure 3, Figure 4 and Figure 5; after esterification with methanol to form volatile methyl esters) and FT-IR (Figure 6). 

According to the authors [28], the oxidation product obtained using 5 equiv. of hydrogen peroxide contained carboxylic acids with an odd number of carbon atoms (mostly C27 to C39). The main component was carboxylic acid C_28_H_57_COOH, which formed due to oxidative cleavage of C_30_H_60_ α-olefin. They observed only small amounts of short-chain carboxylic acid.

In contrast, the product obtained by oxidation using only oxygen contained acids with even and odd numbers of carbons atoms and more components with lower volatility (number of carbon atoms < 25) (Figure 3; Table 8, Entry 3). This is the effect of free-radical oxidation in which generated peroxy radicals may have abstracted hydrogens from alkyl chain or added to double bonds. Hydrogen abstraction may lead to alkyl chain degradation and the formation of low-molecular-weight products.

Chromatograms of products obtained by the 2-stage method are presented in Figure 4 and Figure 5 (Table 8, Entry 1). The results show that during the second stage of oxidation using O_2_, the amount of C_28_H_57_COOH increased, which confirmed that oxygen partially replaced H_2_O_2_ during the oxidative cleavage of alkenes. Compounds formed during the first steps of oxidation using H_2_O_2_, e.g., diols, may be oxidized to the respective acids via carbon-carbon bond cleavage; however, compared with the product obtained by one-stage oxidation using 5 equiv. of H_2_O_2_, a larger amount of acids with carbon numbers below 25 were obtained, both with even and odd numbers. This was a result of the abovementioned free-radical oxygen oxidation. 

Figure 6 compares the FT-IR spectra of raw materials and products obtained by the two-step method, after the 1st stage of oxidation using 2 equiv. of H_2_O_2_ and the 2nd stage using O_2_. In the spectra of both oxidation products, no C=C band (1640 cm^−1^, stretching vibration) was observed, while bands at 1720 cm^−1^ due to carbonyl groups (C=O) appeared. 

## 4. Conclusions

Studies on the two-step oxidation of long-chain terminal olefins using environmentally-friendly oxidants H_2_O_2_ and O_2_ were performed. In the first stage, C30+ olefins were oxidized using H_2_O_2_. It was established that to obtain a product with a low double bond content, it was beneficial to carry out the reaction using 2 equiv. of H_2_O_2_ in the presence of 0.75 wt.% H_2_WO_4_, 1.5 wt.% Luviquat, 0.1 mL 0.5 M H_2_SO_4_, for 1–2 h at 85 °C. The use of 1 equiv. of H_2_O_2_—according to the reaction stoichiometry sufficient for complete epoxidation of the alkene—resulted in the incomplete conversion of alkenes. This was due to the consumption of hydrogen peroxide in the subsequent oxidation reactions, as well as its partial decomposition. A product with an acid number of 11 and a saponification number of 19 was obtained.

Further oxidation of this product was conducted in the presence of a catalytic system composed of NHPI and Co(II) under a pressurized oxygen atmosphere. The results showed that during the process, the acid and ester contents increased, and a product with an acid number of 29 and a saponification number of 64 was obtained.

A comparison of the product’s composition obtained after the 1st and 2nd reaction stages using GC-MS showed that H_2_O_2_ could be partially replaced by oxygen in oxidative cleavage of long chain α-olefins. It was demonstrated that the content of acids formed by the oxidative cleavage of alkenes increased in the second stage; however, the use of oxygen led to the formation of larger amounts of less-volatile compounds due to alkyl chain degradation.

## Figures and Tables

**Table 1:** Effect of amount and concentration of H_2_O_2_ on the C30+ olefins oxidation.

Entry	Equiv. of H_2_O_2_	H_2_O_2_, %	AN, mg KOH/g	SN, mg KOH/g	EN *, mg KOH/g	C=C, mmol/g	Conversion, %	Viscosity at 120 °C mPa·s
1	1	50	16	22	6	0.782	61	9.7
2	2	50	21	33	13	0.217	89	13.4
3	3	50	25	45	20	0.058	97	10.2
4	4	50	37	57	20	0.033	98	13.4
5 ^a^	1	50	12	15	3	0.782	61	7.1
6 ^a^	2	50	20	41	21	0.195	90	14.9
7 ^a^	3	50	25	44	19	0.095	95	13.9
8 ^a^	4	50	33	49	16	0.080	96	14.2
9 ^a^	1	30	10	21	11	0.656	68	9.1
10 ^a^	2	30	15	26	11	0.332	84	11.7
11 ^a^	3	30	25	43	18	0.108	95	13.4
12 ^a^	4	30	18	31	13	0.207	90	7.6
13 ^b^	1	50	8	17	9	0.915	55	8.2
14 ^b^	2	50	20	37	17	0.397	80	11.1
15 ^b^	3	50	16	21	5	0.066	97	12.3
16 ^b^	4	50	34	60	26	0.037	98	10.8

Note: Olefins C30+ 40 g, H_2_O_2_ 50 or 30%, H₂WO₄ 3 wt.%, Luviquat 5 wt.%, H_2_SO_4_ 0.5M 0.1 mL, 85 °C, 10 h, 400 rpm, ^a^ H₂WO₄ 1.5 wt.%, Luviquat 2.5 wt.%, ^b^ solvent 32 g, 300 rpm.; * EN—Ester number.

**Table 2:** Effect of H_2_WO_4_ and Luviquat on the C30+ olefins oxidation using H_2_O_2_.

Entry	Luviquat, wt.%	H_2_WO_4_, wt.%	AN, mg KOH/g	SN, mg KOH/g	EN, mg KOH/g	C=C, mmol/g	Conversion, %	Viscosity at 120 °C, mPa·s
1	-	-	5	11	6	1.816	11	3.5
2	-	1.5	4	8	4	0.879	57	7.1
3	2.5	-	9	25	16	1.344	34	4.9
4	5.0	-	6	5	1	1.927	5	3.7
5	0.75	1.5	16	26	10	0.236	88	4.9
6	1.5	1.5	18	38	20	0.121	94	3.9
7	2.5	1.5	25	44	19	0.095	95	13.9
8	5.0	1.5	26	40	14	0.050	98	10.0
9	2.5	0.75	21	42	21	0.227	89	6.6
10	5.0	3.0	25	45	20	0.058	97	10.2

Note: Olefins C30+ 40 g, 3 equiv. of H_2_O_2_ 50%, H_2_SO_4_ 0.5M 0.1 mL, 85 °C, 10 h, 400 rpm.

**Table 3:** Effect of H_2_SO_4_ or H_3_PO_4_ on the C30+ olefins oxidation using H_2_O_2_.

Entry.	Acid 0.5M, mL	AN, mg KOH/g	SN, mg KOH/g	EN, mg KOH/g	C=C, mmol/g	Conversion, %	Viscosity at 120 °C, mPa·s
1^a^	-	15	27	12	0.205	90	8.5
2^a^	0.1	18	39	21	0.195	90	14.9
3^a^	1	10	28	18	0.237	88	8.5
4^b^	0.1	22	39	17	0.233	89	12.6
5^b^	1	17	32	15	0.229	89	15.6

Note: Olefins C30+ 40 g, 2 equiv. of H_2_O_2_ 50%, H₂WO₄ 1.5 wt.%, Luviquat 2.5wt.%, 85 °C, 10 h, 400 rpm, ^a^ H_2_SO_4_, ^b^ H_3_PO_4._

**Table 4:** Effect of reaction time on the C30+ olefins oxidation using H_2_O_2_.

Entry.	Luviquat, wt.%	H_2_WO_4_, wt.%	Time, h	AN, mg KOH/g	SN, mg KOH/g	EN, mg KOH/g	C=C, mmol/g	Conversion, %	Viscosity at 120 °C, mPa·s
1	0.75	0.75	2	12	23	11	0.333	84	11.7
2	0.75	0.75	4	14	27	13	0.247	88	11.6
3	0.75	1.5	2	17	29	12	0.346	83	11.6
4	0.75	1.5	4	14	28	14	0.506	75	10.5
5	1.5	0.75	2	18	32	14	0.207	90	11.5
6	1.5	0.75	4	12	32	20	0.195	90	11.4
7	1.5	1.5	2	15	30	15	0.256	87	12.1
8	1.5	1.5	4	16	36	20	0.254	87	12.1
9	2.5	1.5	2	16	30	14	0.178	91	11.9
10	2.5	1.5	4	20	31	11	0.227	89	9.2
11	2.5	1.5	6	17	30	13	0.201	90	9.1
12	2.5	1.5	8	21	35	14	0.145	93	8.2
13	2.5	1.5	10	18	39	21	0.195	90	14.9

Note: Olefins C30+ 40 g, H_2_O_2_ 50% - 2 equiv., H_2_SO_4_ 0.5 M 0.1 mL, 85 °C, 10 h, 400 rpm.

**Table 5:** Properties of pre-oxidized with H_2_O_2_ raw material used in the oxidation reaction using oxygen.

Entry.	Melting point, °C	AN, mg KOH/g	SN, mg KOH/g	EN, mg KOH/g	C=C, mmol/g	Conversion, %	Viscosity at 120 °C, mPa·s
1	85.1–91.0	31	50	19	0.030	99	13.1

Note: Olefins C30+ 200 g, H_2_O_2_ 50% 3 equiv., H₂WO₄ 3.0 wt.%, Luviquat 5.0 wt.%, H_2_SO_4_ 0.5 M 0.5 mL, 85 °C, 10 h, 400 rpm.

**Table 6:** Effect of catalyst on the oxidation of the pre-oxidized raw material using oxygen.

Entry.	Catalyst	Melting Point, °C	AN, mgKOH/gL	SN, mgKOH/g	EN mgKOH/g	Viscosity at 120 °C, mPa·s
1	-	81.0–87.9	27	45	18	14.1
2	Co(II)	74.3–85.7	55	111	56	16.3
3	NHPI	77.6–90.5	42	66	24	16.1
4^a^	Co(II)/NHPI	70.9–86.1	42	95	53	14.3
5	Co(II)/NHPI	70.8–85.8	71	132	61	16.5
6^b^	Co(II)/NHPI	58.2–79.1	59	111	53	16.9

Note: Raw material (described in Table 5) 15 g, Co(acac)_2_ 1 wt.%., NHPI 1 wt.%, 100 °C, 0.5 MPa O_2_, 5 h, 1000 rpm ^a^ Co(II) 0.1 wt.%, ^b^ air flow 3 L/h, 0.1 MPa,.

**Table 7:** Effect of temperature and reaction time on the oxidation of the pre-oxidized raw material using oxygen.

Entry.	Temperature, °C	Time, h	Melting Point, °C	AN, mgKOH/g	SN, mgKOH/g	EN, mgKOH/g	Viscosity at 120 °C, mPa·s
1	90	5	73.6–85.7	65	105	40	17.1
2	100	2.5	73.7–85.4	70	117	47	16.3
3	100	5	70.8–85.8	71	132	61	16.5
4	100	7.5	66.8–76.8	82	140	58	17.0
5	110	5	68.4–81.8	74	132	58	17.6

Note: raw material (described in Table 5) 15 g, Co(acac)_2_ 1 wt.%, NHPI 1 wt.%, 0.5 MPa O_2_, 1000 rpm.

**Table 8:** Products of C30+ olefin oxidation using H_2_O_2_, O_2_, or both H_2_O_2_ and O_2._

Entry.	Oxidant	Amount of Oxidant	Melting Point, °C	AN, mgKOH/g	SN, mgKOH/g	EN, mgKOH/g	Viscosity at 120 °C, mPa·s
1 ^a^ 1 ^b^	H_2_O_2_ O_2_	2 equiv. 0.5 MPa	78.4–86.4 59.0–68.7	11 29	19 64	8 35	9.2 11.3
2 [28] ^c^	H_2_O_2_	5 equiv.	nd	59	75	16	nd
3^d^	O_2_	0.5 MPa	55.2–68.4	75	135	60	12.7

^a^ 1st stage: olefins C30+ 40 g, H_2_O_2_ 50%, H₂WO₄ 0.75 wt.%, Luviquat 1.5 wt.%, H_3_PO_4_ 0.5 M 0.1 mL (0.05 mmol), 85 °C, 2 h, 400 rpm; ^b^ 2nd stage: product of 1st stage—15 g; Co(acac)_2_ 0.1 wt.%, NHPI 1wt.%, 0.5 MPa O_2_, 100 °C, 5 h, 1000 rpm; ^c^ Olefins C30+ 40 g, H_2_O_2_ 50%, H₂WO₄ 1.5 wt.%, Luviquat 5.0 wt.%, H_2_SO_4_ 1M 17 μmol/g, 90 °C, 10 h, 500 rpm [28]; ^d^ Olefins C30+ 15 g, Co(acac)_2_ 0.1 wt.%, NHPI 1 wt.%, 0.5 MPa O_2_, 100 °C, 5 h, 1000 rpm.

## Supplementary Materials

The following are available online at https://www.mdpi.com/1996-1944/13/20/4545/s1, Figures S1–S8: Mass spectra of olefins identified in starting material, Figures S9–S12: Mass spectra of esters identified in oxidation products.
