# Supplementary material for: Oxidation of Long-Chain α-Olefins Using Environmentally-Friendly Oxidants
Source: Materials (Basel). 2020 Oct 13;13(20):4545. doi: 10.3390/ma13204545 (PMC7602050; doi:10.3390/ma13204545)
Supplement: Supplementary file 1 [file materials-13-04545-s001.pdf]

materials

Supplementary Materials

Oxidation of Long-Chain α-Olefins Using Environmentally-friendly Oxidants

Kamil Peckh, Dawid Lisicki, Gabriela Talik and Beata Orlińska *

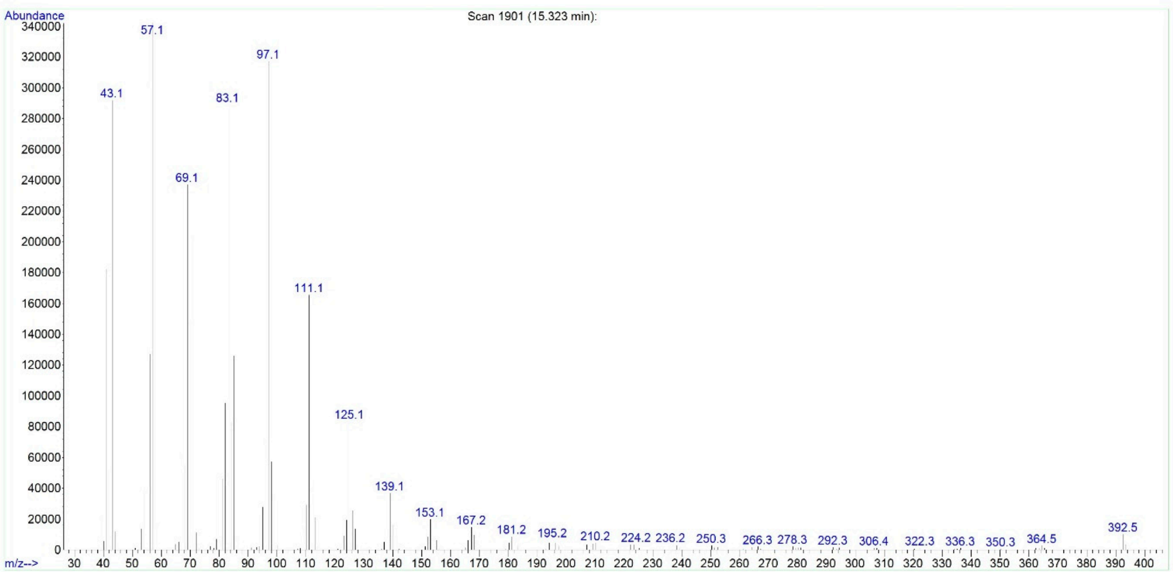

Figure S1. Mass Spectrum of $C_{28}H_{56}$.

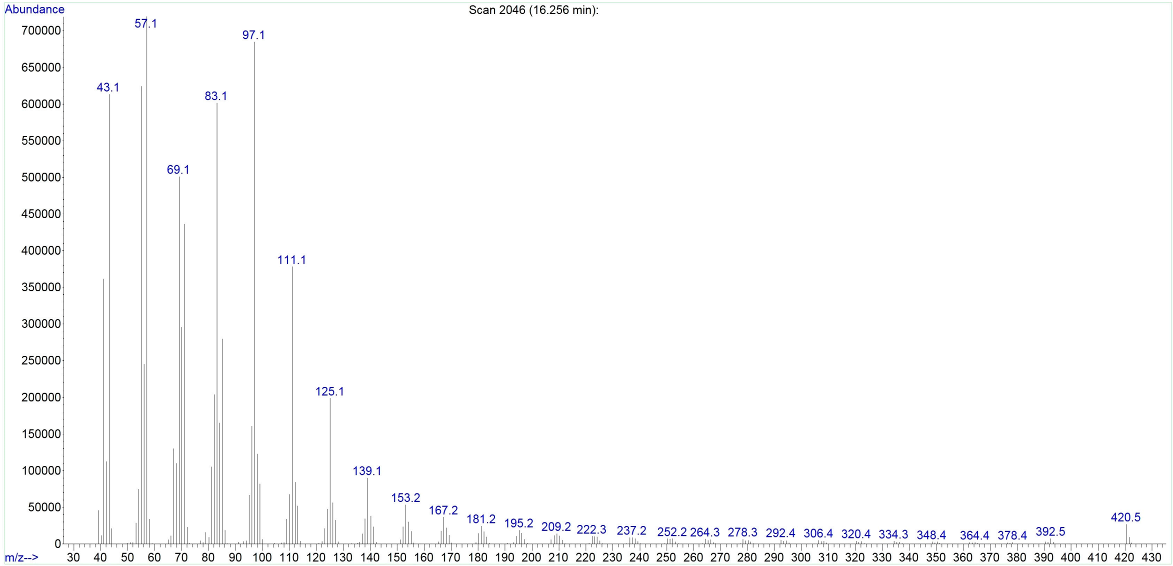

Figure S2. Mass Spectrum of $C_{30}H_{60}$.

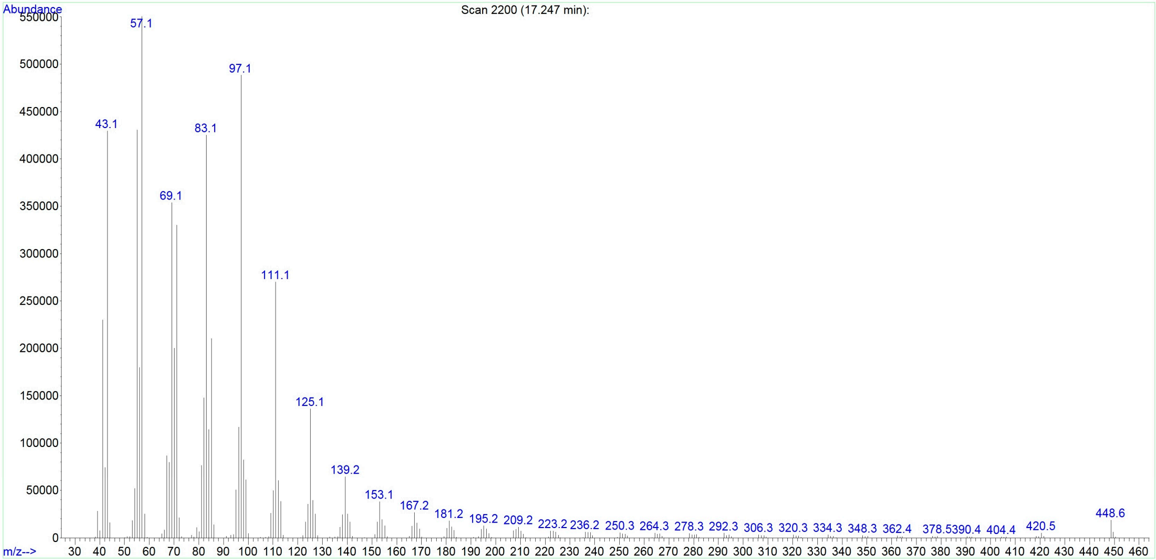

Figure S3. Mass Spectrum of $C_{32}H_{64}$

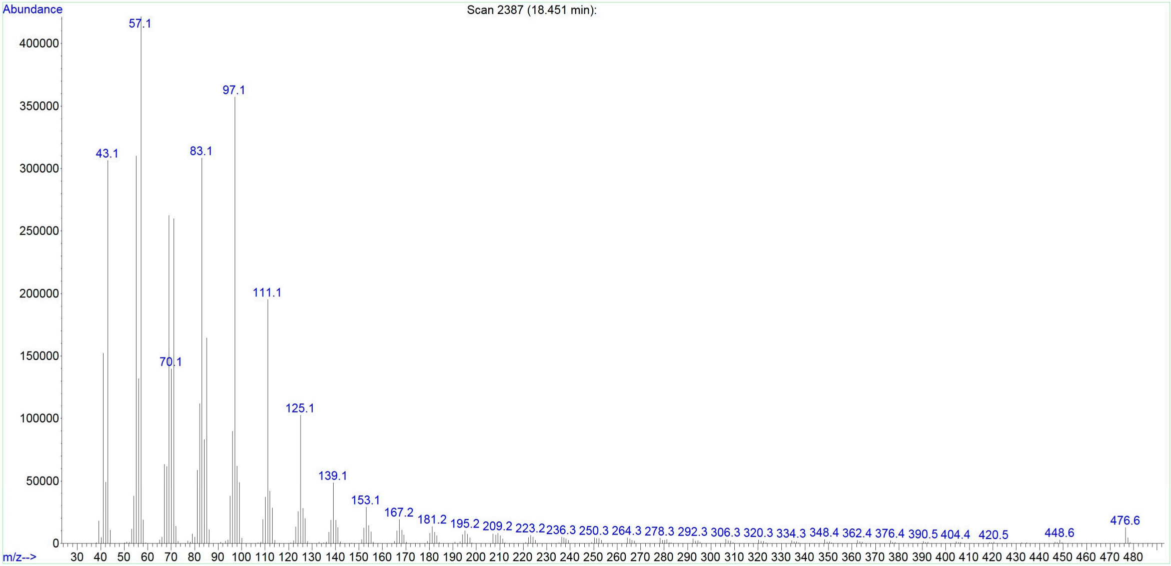

Figure S4. Mass Spectrum of $C_{34}H_{68}$.

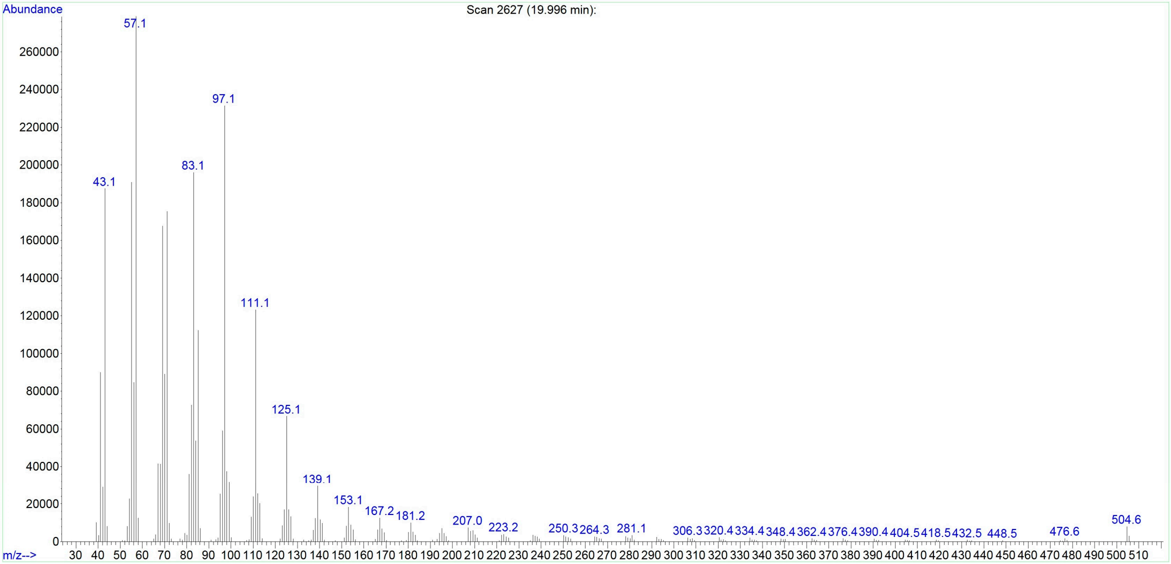

Figure S5. Mass Spectrum of $C_{36}H_{72}$.

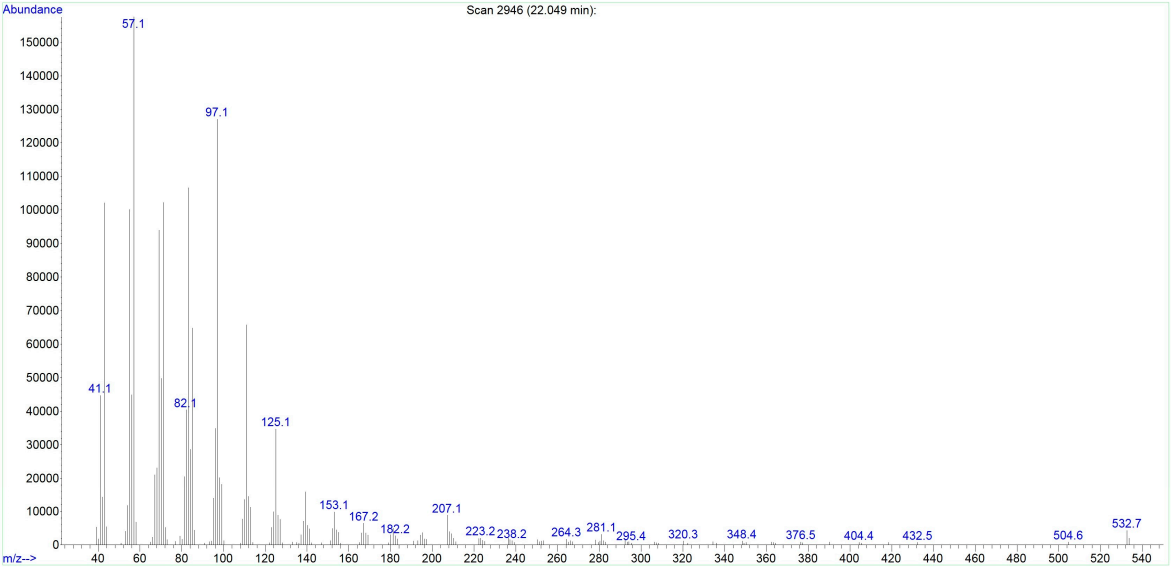

Figure S6. Mass Spectrum of $C_{38}H_{76}$.

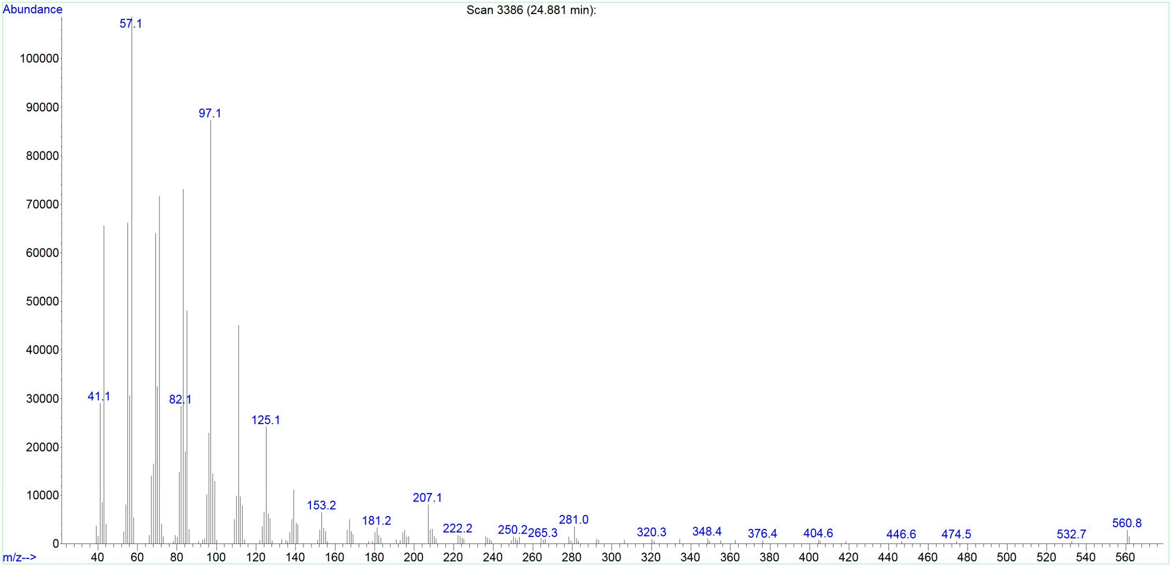

Figure S7. Mass Spectrum of $C_{40}H_{80}$.

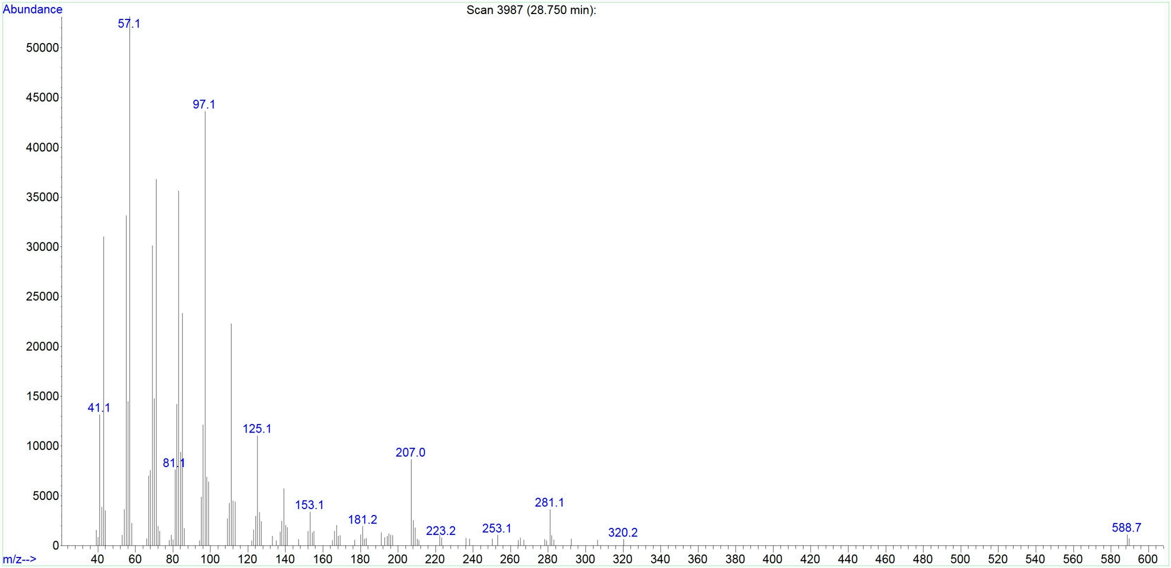

Figure S8. Mass Spectrum of $C_{42}H_{84}$.

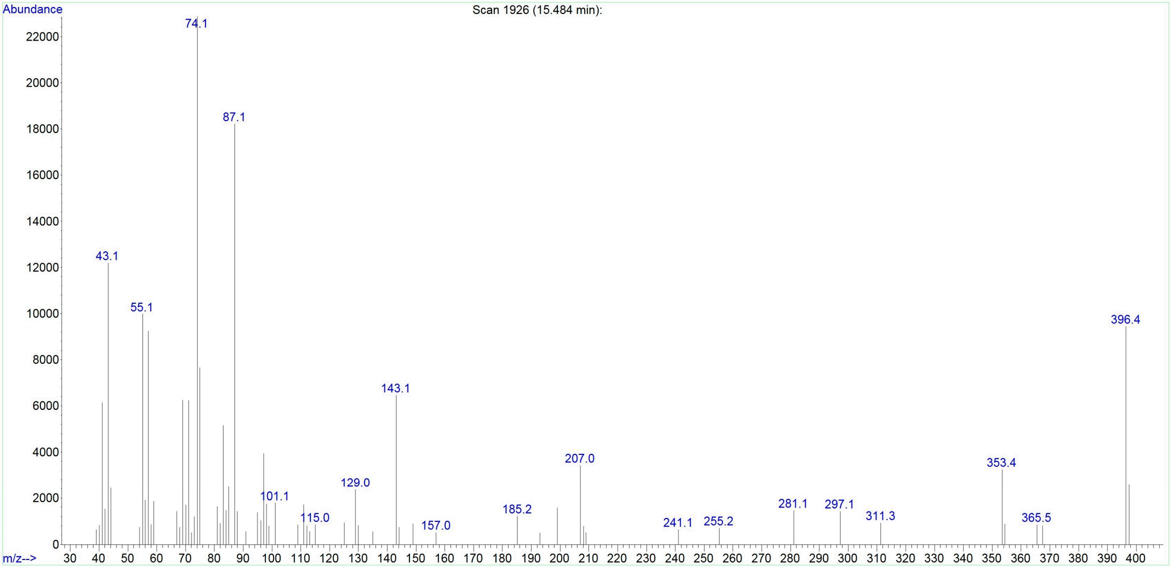

Figure S9. Mass Spectrum of $C_{24}H_{49}COOCH_3$.

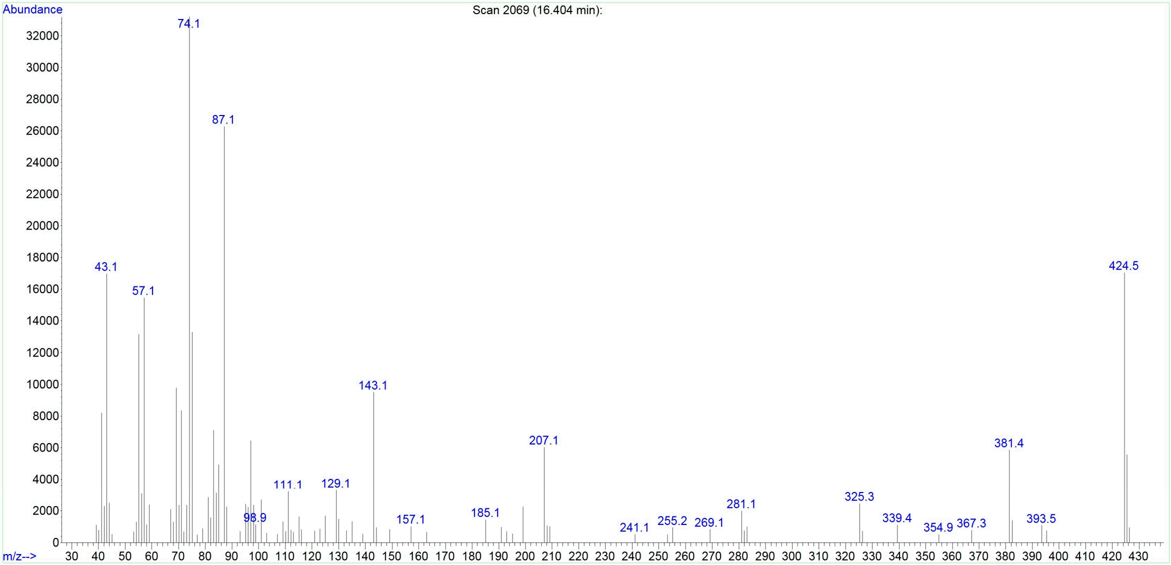

Figure S10. Mass Spectrum of $C_{26}H_{53}COOCH_3$.

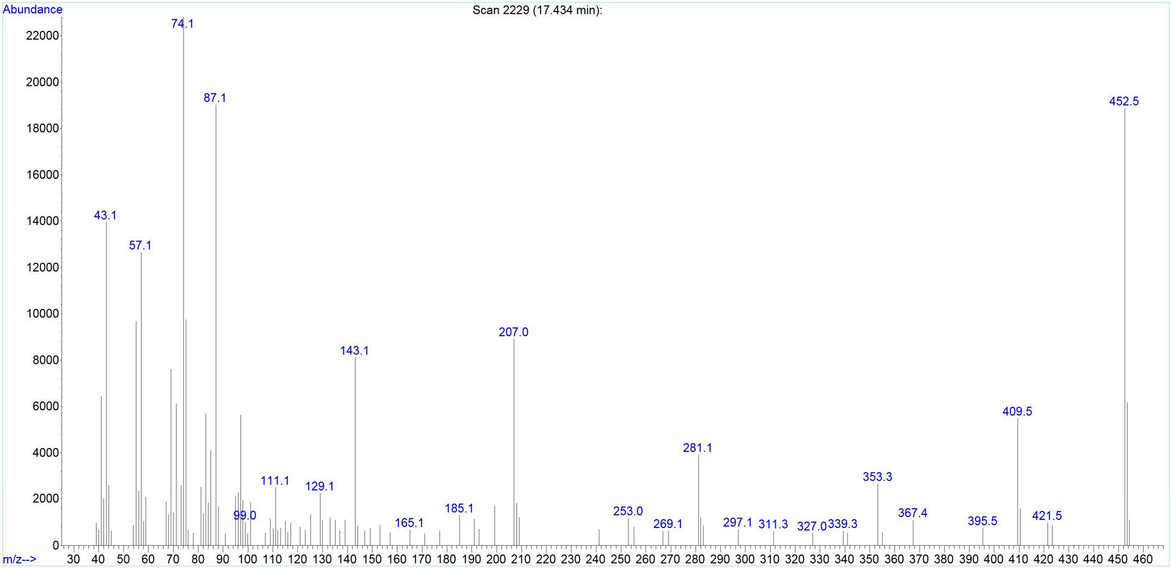

Figure S11. Mass Spectrum of $C_{28}H_{57}COOCH_3$.

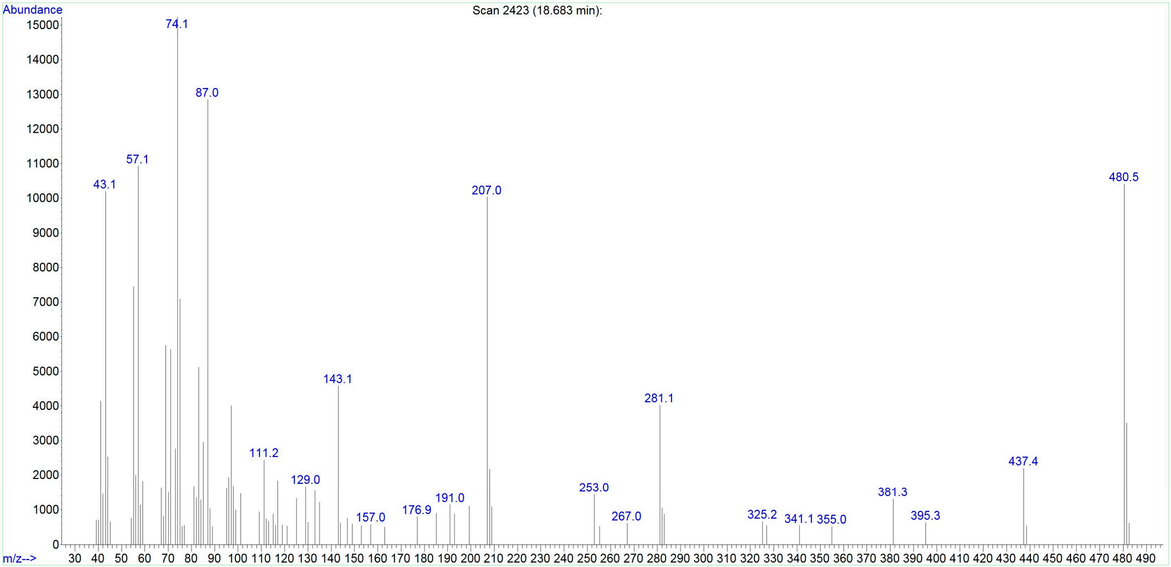

Figure S12. Mass Spectrum of $C_{30}H_{61}COOCH_3$.
